# Does the liver talk to the brain? If so, how and why?

**DOI:** 10.1371/journal.pbio.3003491

**Published:** 2025-11-13

**Authors:** Young-Hwan Jo

**Affiliations:** 1 The Fleischer Institute for Diabetes and Metabolism, Albert Einstein College of Medicine, New York, New York, United States of America; 2 Division of Endocrinology, Department of Medicine, Albert Einstein College of Medicine, New York, New York, United States of America; 3 Department of Molecular Pharmacology, Albert Einstein College of Medicine, New York, New York, United States of America; 4 Department of Neuroscience, Albert Einstein College of Medicine, New York, New York, United States of America

## Abstract

Interoceptive signals from visceral organs, such as heart, lungs and gut, are known to influence many aspects of organismal physiology and behavior. This Perspective argues that liver signaling via the vagal nerve is another avenue of body-brain communication that shapes metabolism and mood.

## Teaser

The liver communicates with the brain by transmitting interoceptive signals, including nutrients, hormones, and cytokines, potentially via the liver-innervating vagal sensory neurons. This communication is crucial for maintaining metabolic homeostasis and emotional stability.

The liver, the largest organ in the body, has a crucial role in various essential physiological processes. In the fasted state, hepatic glucose production accounts for a substantial portion of the endogenous glucose output in humans; as glucose is the primary energy source in the brain, its proper function therefore depends substantially on hepatic glucose synthesis. While insulin and glucagon (produced by the pancreas) control hepatic glucose production in response to nutrient levels, the brain also significantly influences hepatic glucose output via the autonomic nervous system [1]. Contrary to the traditional top-down neural regulatory mechanism, emerging evidence suggests that peripheral organs, such as the liver and gastrointestinal tract, can directly influence brain function through interoception, which involves bidirectional signal processing between the brain and internal organs. However, despite extensive research on the gut–brain axis in relation to energy metabolism and mental health, the liver–brain axis has received limited attention.

So, what are hepatic interoceptive signals? The liver comprises diverse cell types that produce different interoceptive signals. Hepatocytes have metabolic roles, but also serve endocrine functions by producing and releasing hepatokines and bile acids. Cholangiocytes, located along the bile ducts, further modify and transport bile acids, contributing to bile composition. Kupffer cells respond dynamically to environmental stimuli by secreting either pro-inflammatory or anti-inflammatory cytokines. Additionally, the portal vein transports nutrients, such as glucose, fatty acids, and amino acids, which are absorbed from the gastrointestinal tract. It also carries molecular cues, such as osmolarity changes and endotoxins. Together, these molecules and cues constitute hepatic interoceptive signal molecules that are transmitted to the brain. They reflect the functional status of the liver, including its capacity for nutrient processing, energy storage, and involvement in pathological conditions such as inflammation, metabolic dysfunction, and liver disease. However, longstanding controversies exist around exactly how these signals reach the brain, particularly regarding the presence of hepatic vagal afferent and efferent nerves. Despite some findings to the contrary, I would argue that the liver signals to the brain via the vagal nerve, and that this is relevant to the metabolic and emotional state of the individual.

In the late 1920s, Dr Kuntz described that the sugar center in the medulla oblongata, where the dorsal motor nucleus of the vagus is situated, consistently received afferent impulses, particularly via the vagi, and had a significant role in the regulation of hepatic glucose production [2]. Vagal sensory nerves were then identified within hepatic lobules and connective tissue in dogs and humans in a histochemical study in the late 1950s [3]. Histochemical staining using cholinesterase activity in the 1960s revealed potential parasympathetic cholinergic nerve fibers in the liver parenchyma, hepatic vessels, and bile ducts of guinea pigs [4]. Later, a network of cholinesterase-positive nerve fibers was demonstrated within the lobules, closely associated with hepatocytes and sinusoids in both rats [5] and humans [6]. A more focused histochemical investigation of the nerves of the hepatic branch of the vagus nerve in the 1980s revealed central projection sites within the dorsal vagal complex specific to vagal afferent and efferent nerves in rats [7]. In addition to these early histochemical studies, numerous animal studies utilizing hepatic branch denervation have demonstrated the significant roles of hepatic vagal afferent and efferent nerves in regulating liver regeneration, inflammation, hepatic glucose production, insulin resistance, and liver-visceral organ communication [8,9]. These previous studies provide substantial evidence for the existence of vagal sensory and parasympathetic cholinergic innervation of the liver.

My lab recently demonstrated the presence of vagal sensory and parasympathetic cholinergic inputs in the mouse liver [10,11], confirming the results of these earlier studies [2–7]. Using both anterograde and retrograde adeno-associated viral tracers, we successfully labeled vagal afferent and efferent nerve fibers in the liver. Advillin-positive vagal sensory neurons projected their axons predominantly to the periportal areas [11], suggesting their potential role in sensing and transmitting interoceptive signaling molecules to the brain. Additionally, parasympathetic preganglionic cholinergic nerves innervated the bile ducts and a small subset of hepatocytes [10]. These findings raise an important question: can this limited population of hepatocytes receiving cholinergic input generate a physiologically meaningful output? Due to liver zonation, hepatocytes in distinct zones of hepatic lobules exhibit specialized metabolic functions. More importantly, hepatocytes seem to communicate through gap junctions, facilitating direct intercellular communication and coordination of metabolic activities. In this context, even a limited population of cholinergic neurons and nerves could exert a substantial influence on liver function, as localized cholinergic signaling may propagate through hepatocytes to modulate broad metabolic functions.

**Fig 1:**
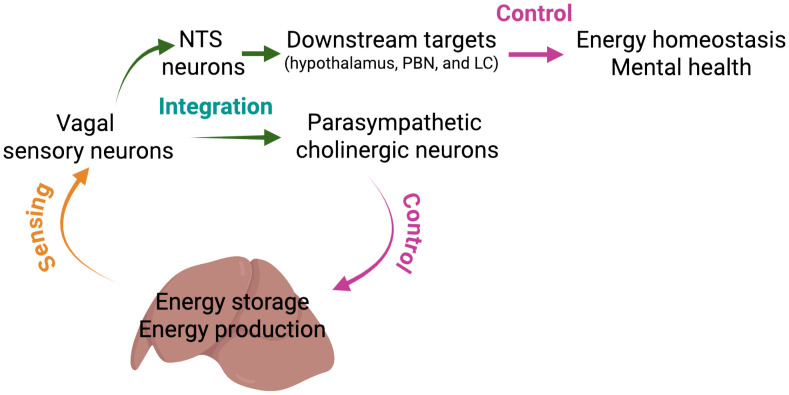
Proposed model of liver interoceptive signaling. Liver-innervating vagal sensory neurons are capable of detecting hepatic interoceptive signaling molecules. This interoceptive information directly transmits to neurons in the nucleus tractus solitarius (NTS) region of the brainstem, which serves as a central integrative hub. Signals are relayed to the arcuate nucleus of the hypothalamus, the parabrachial nucleus (PBN), and the locus coeruleus (LC), which are implicated in the regulation of energy homeostasis, stress responses and emotional behavior. Additionally, the dorsal motor nucleus of the vagus (DMV) receives direct synaptic input from liver-innervating vagal sensory neurons. Parasympathetic cholinergic neurons modulate liver functions, including energy production, nutrient storage, and metabolic adaptation, so together, these bidirectional neural pathways form a dynamic liver–brain axis that could have a critical role in maintaining metabolic homeostasis and emotional stability. The figure is created in BioRender. Jo, Y. (2025) https://BioRender.com/xl042u4.

Hepatic interoception may also have an effect on liver function and energy metabolism. Given that the liver is responsible for the production and storage of energy, circadian rhythms in the liver are essential for maintaining energy balance. Interestingly, high-fat diet (HFD) feeding disrupts the circadian rhythm in the liver, causing changes to food intake patterns and resulting in an increase in body weight in mice [12]. Surprisingly, deleting liver-innervating vagal sensory neurons completely prevents aberrant food intake patterns, and hepatic vagotomy protects against body weight gain during HFD feeding [12], suggesting the importance of hepatic interoception at the vagal sensory level in food intake and energy homeostasis. Work from my lab has additionally shown that the selective deletion of liver-innervating vagal afferent and efferent nerves completely prevents the onset of diet-induced hepatic steatosis in mice [11], suggesting that this reciprocal communication mediated by liver-innervating afferent and efferent neurons may have a pivotal role in energy storage in response to nutrient availability (Fig 1). Additionally, loss of liver-innervating advillin-positive vagal sensory neurons results in diminished anxiety-like behavior under obesogenic conditions [11]. These findings highlight the idea that the brain relies on precise and sufficient interoceptive signals, particularly via the vagal sensory neural pathway, to maintain emotional and metabolic homeostasis (Fig 1).

Nonetheless, the presence of vagal nerve innervation to the liver, particularly at the level of the hepatic lobules, remains a subject of ongoing scientific debate. Two 3D imaging studies have demonstrated abundant tyrosine hydroxylase (TH)-positive sympathetic nerves within the livers of mice, monkeys, and humans, and yet failed to detect any nerve fibers positive for cholinergic neuron markers [13,14]. This discrepancy may reflect anatomical differences in TH and vescicular acetylcholine transporter (VAChT) expression across somas, axons, and terminals. TH produces a continuous and clearly delineated staining along the catecholaminergic fibers. However, immunostaining with an antibody against VAChT, which is commonly used to label cholinergic axons and nerve terminals, always yields puncta-like staining, as the VAChT is present exclusively in the synaptic vesicles of cholinergic nerve terminals rather than along the entire axonal shaft. Moving forward, it is essential to re-examine the cholinergic system in the liver to gain a better understanding of how the autonomic nervous system of the liver controls metabolic homeostasis. Future studies integrating advanced neural tracing and functional assays will be pivotal in unraveling this complex liver–brain axis. For example, assays targeting cholinergic activity, such as optogenetic and chemogenetic stimulation, combined with acetylcholine biosensors and calcium imaging, may offer compelling evidence for the presence and physiological significance of parasympathetic cholinergic innervation of the liver.

Overall, these insights underscore the need for future studies to explore the functional significance of bidirectional liver–brain crosstalk, as this may have a critical role in regulating metabolic and emotional homeostasis. Understanding the presence and role of hepatic vagal innervation, particularly the parasympathetic cholinergic input, is crucial for elucidating how interoceptive signals influence systemic physiology.

## Abbreviations

HFD: high-fat diet
LC: locus coeruleus
NTS: nucleus tractus solitarius
PBN: parabrachial nucleus
TH: tyrosine hydroxylase
VAChT: vesicular acetylcholine transporter
